# Supplementary material for: Automated Ex Situ Assays of Amyloid Formation on a Microfluidic Platform
Source: Biophys J. 2016 Feb 2;110(3):555–60. doi: 10.1016/j.bpj.2015.11.3523 (PMC4744157; doi:10.1016/j.bpj.2015.11.3523)
Supplement: Document S1. Supporting Materials and Methods and Figs. S1–S4 [file mmc1.pdf]

Automated *ex situ* aggregation assays - Supporting Material

K.-L. Saar, E. V. Yates, T. Müller, S. Saunier, C. M. Dobson and
T. P. J. Knowles

1 Pre-formed insulin fibrils

In order to accelerate the aggregation process 5% of the initial bovine insulin was added in the form of preformed fibrils. These fibrils were generated by incubating 10 mg mL^{-1} insulin solution (pH 2, no NaCl) for 24 hours at 60 °C with maximum stirring. Before adding the thus formed seeds to the aggregation mixture, they were sonicated (3 times for 5 minutes, Bandelin Sonopuls HD2070, 8 W, VS 70 probe) in order to fragment the fibrils and hence increase the number of free ends available for aggregation. Figure S1 shows an AFM image of the preformed fibrils.

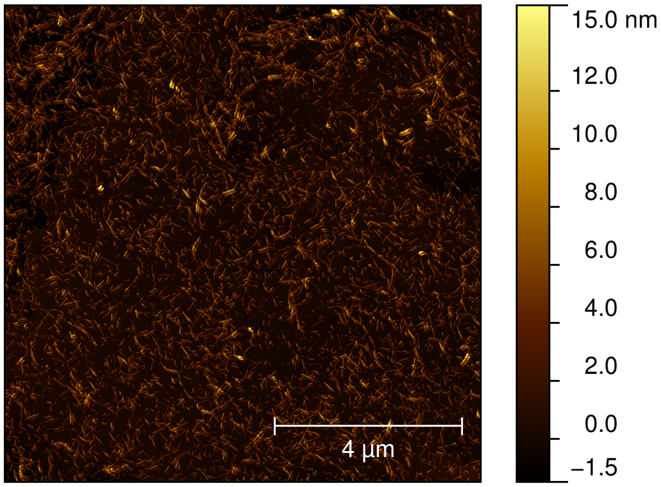

Figure S1. An AFM image of the preformed seeds that were added to the aggregation assay in order to accelerate the aggregation process.

2 Non-linear scaling in *in situ* assays

As demonstrated in Figure 1b (main text), linear scaling of fluorescence intensity with bovine insulin aggregate concentration appears to be a general trend of *ex situ* assays within the concentration range of protein studied. However, such linear scaling is not observed for *in situ* assays under the same aggregation conditions. We investigated further whether the absence of such scaling is specific to the conditions used in these experiments or a more general trend that is occurring in other of the buffer condition and other concentration of dye. Indeed, Figure S2 illustrates the absence of a simple scaling relationship between the fluorescence intensity and the endpoint concentration of bovine insulin fibrils in 50 mM and 100 mM NaCl buffers (both at pH 2.0) followed at ThT concentrations of 2 μM and 20 μM ThT.

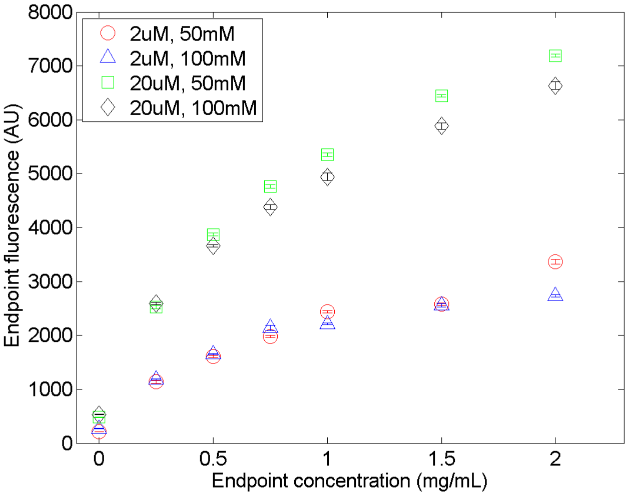

Figure S2. Absence of simple linear scaling between fluorescence intensity and insulin fibril concentration in *in situ* assays aggregating in 50 mM and 100 mM NaCl buffers (both at pH 2.0) followed at ThT concentrations of 2 μM and 20 μM. Linear scaling is observed in *ex situ* assays as demonstrated in Figure 1b of the main text of the Article.

3 Device design

In order to guide the design of the device the following calculations were performed.

By the time a section of fluid reaches the measurement point the dye should have fully diffused into the protein stream. For example, in an SL device, ThT needs to diffuse by

$$x = \frac{1}{2} \times w_{protein} = \frac{1}{2} \times w_{total} \times \frac{Q_{protein}}{Q_{total}} = 0.5 \times 400\mu m \times \frac{50\ \mu L\ h^{-1}}{(200+50)\ \mu L\ h^{-1}} = 40\mu m \quad (1)$$

where the factor $\frac{1}{2}$ arises from symmetry and $w_{protein}$ and w_{total} represent the width of the protein stream in the measurement area and that of the measurement channel, respectively. Q_i's correspond to the respective flow rates.

Hence, it takes

$$t_{ThT} \sim \left(\frac{x^2}{2 \times D_{ThT}}\right) \sim \left(\frac{(40 \times 10^{-6})^2\ m^2}{2 \times 8 \times 10^{-10}\ m^2\ s^{-1}}\right) \sim 1s \quad (2)$$

for the ThT to diffuse into the protein stream.

Finally, in a rectangular duct the centreline velocity of the fluid can be approximated to be:

$$u_{CL} = \frac{Q_{total}}{A} = \frac{\frac{250\ mm^3}{h} \times \frac{1\ h}{3600\ s}}{400 \times 10^{-3}\ mm \times 100 \times 10^{-3}\ mm} = 1.7\ mm\ s^{-1} \quad (3)$$

The measurement was taken around 14 mm downstream of the point where the fluids first mix to ensure complete mixing of the ThT molecules with the fibrils.

4 Fluorescence intensity measurements and calculations

As explained in the previous section the fluorescence intensity at each time point was recorded at a point sufficiently downstream for the ThT molecules to have fully diffused into the protein stream. The recorded values were calculated as the difference between the fluorescence intensity of the ThT bound to fibrils (centre of the channel; the average value across the blue rectangle with area of 600×30 pixels) and that of the non-bound form (edges of the channel, the average value across the two brown rectangles each with area of 600×200 pixels) as illustrated in Figure S3. Averaging across the rectangular shapes allows eliminating any possible effects that could have arisen from non-uniform illumination of the field of view.

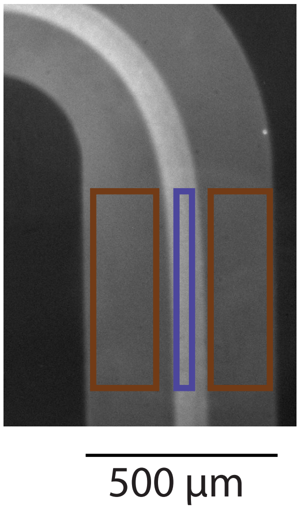

Figure S3. The fluorescence intensity at each time point was calculated as the difference between the fibril-bound form of the ThT (centre of the channel) and its non-bound form (edges of the channel). Both values were averaged across rectangular shapes in order to eliminate any variations from potential non-uniform illumination of the imaging area.

5 Adhesion in SL devices at higher concentrations of protein

The SL device could be effectively used for following protein aggregation at lower protein concentrations. However, at higher concentrations adhesion of the protein to the PDMS surface was observed. For instance, Figure S4 shows the adhesion in a SL device at protein fibril concentration of 0.2 mg mL^{-1} after 2 hours. Hence, ML devices were design and fabricated to

eliminate any interaction between protein stream and the walls of the microfluidic channel by flaking the protein stream by co-flowing buffer from all directions (see main text for details).

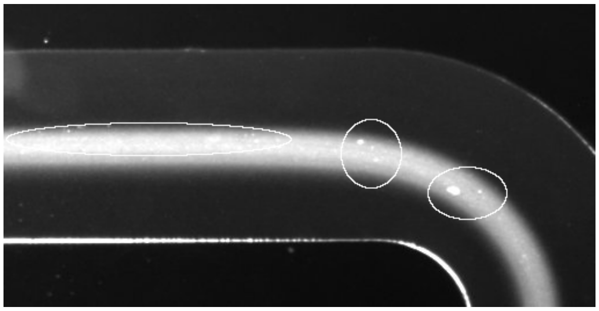

Figure S4. Fibril sticking to the PDMS surface of the channels at protein concentration of 0.2 mg mL^{-1} after 2 hours.
