# Supplementary material for: Automated Ex Situ Assays of Amyloid Formation on a Microfluidic Platform
Source: Biophys J. 2016 Feb 2;110(3):555–60. doi: 10.1016/j.bpj.2015.11.3523 (PMC4744157; doi:10.1016/j.bpj.2015.11.3523)
Supplement: Document S2. Article plus Supporting Material [file mmc2.pdf]

Biophysical Journal Volume 110 February 2016 555–560

Article

Automated Ex Situ Assays of Amyloid Formation on a Microfluidic Platform

Kadi-Liis Saar,[1] Emma V. Yates,[1] Thomas Müller,[1] Séverine Saunier,[1] Christopher M. Dobson,[1] and Tuomas P. J. Knowles[1,*]
[1]Department of Chemistry, University of Cambridge, Cambridge, United Kingdom

ABSTRACT Increasingly prevalent neurodegenerative diseases are associated with the formation of nanoscale amyloid aggregates from normally soluble peptides and proteins. A widely used strategy for following the aggregation process and defining its kinetics involves the use of extrinsic dyes that undergo a spectral shift when bound to β-sheet-rich aggregates. An attractive route to carry out such studies is to perform ex situ assays, where the dye molecules are not present in the reaction mixture, but instead are only introduced into aliquots taken from the reaction at regular time intervals to avoid the possibility that the dye molecules interfere with the aggregation process. However, such ex situ measurements are time-consuming to perform, require large sample volumes, and do not provide for real-time observation of aggregation phenomena. To overcome these limitations, here we have designed and fabricated microfluidic devices that offer continuous and automated real-time ex situ tracking of the protein aggregation process. This device allows us to improve the time resolution of ex situ aggregation assays relative to conventional assays by more than one order of magnitude. The availability of an automated system for tracking the progress of protein aggregation reactions without the presence of marker molecules in the reaction mixtures opens up the possibility of routine noninvasive study of protein aggregation phenomena.

INTRODUCTION

The importance of folding polypeptide chains into three-dimensional structures has been recognized across a number of fields, such as medicine, food processing, material science, and nanotechnology (1–7). In particular, protein misfolding, the subsequent amyloid formation, and the eventual buildup of fibrillar deposits, has been brought into connection with the onset of a wide range of neurodegenerative and other disorders, such as Alzheimer's, Parkinson's disease, and type-II diabetes (8–14). Despite the well-established connection between protein aggregation and neurodegenerative disorders, the molecular mechanisms that underlie this process have been challenging to establish. This difficulty has in part originated from the challenges associated with performing reliable assays of this process in vitro. One of the most frequently employed techniques for monitoring of aggregation involves the use of extrinsic amyloidophilic probes. Upon binding to β-sheet-rich fibrils, such dyes exhibit changes in their fluorescent spectra that can be recorded externally with a spectrophotometer, and hence allow the β-sheet-rich amyloid aggregates to be detected and their amount to be quantified (15).

Submitted July 21, 2015, and accepted for publication November 25, 2015.
*Correspondence: tpjk2@cam.ac.uk
This is an open access article under the CC BY license (http://creativecommons.org/licenses/by/4.0/).
Thomas Müller's present address is Fluidic Analytics Ltd., Cambridge, United Kingdom.
Editor: David Eliezer.
© 2016 The Authors
0006-3495/16/02/0555/6

Yet many of the frequently used probes, such as Congo Red or thioflavin S, have a propensity to influence protein misfolding and aggregation processes and do not permit reliable extraction of quantitative information on the concentration of aggregated material present (15–17). Indeed, some very effective β-breaker molecules that inhibit protein aggregation have structures that are modeled on amyloidophilic dyes (18–21). Other probes, such as thioflavin T (ThT), have been suggested not to exhibit any or exhibit only minimal interference with the aggregation process (16,17). However, the exact mechanism of their binding to fibrils is still unclear (17,21,22) and conventional in situ assays (Fig. 1 *a I*) where the marker molecules are present throughout the reaction, commonly yield signals which, as shown in this work (Fig. 1 *b*, *circles*) as well as by others (23,24), are not directly proportional to the concentration of aggregates and are hence challenging to interpret in a quantitative manner. Moreover, the fluorescence properties of ThT, especially its quantum yield, are affected by extrinsic conditions (15), and because the fluorescence readout takes place directly within the reaction environment, the measured signal is highly dependent on the surrounding environment—making comparison between different experimental conditions challenging (Fig. 1 *c*, *circles*).

These problems can be effectively eliminated through the use of an ex situ measurement strategy (Fig. 1 *a II*), where the probe molecules are not brought into contact with the reaction mixture but instead with aliquots taken from the aggregation sample, thus avoiding any interaction between the aggregating protein and the dye. In addition to allowing

http://dx.doi.org/10.1016/j.bpj.2015.11.3523

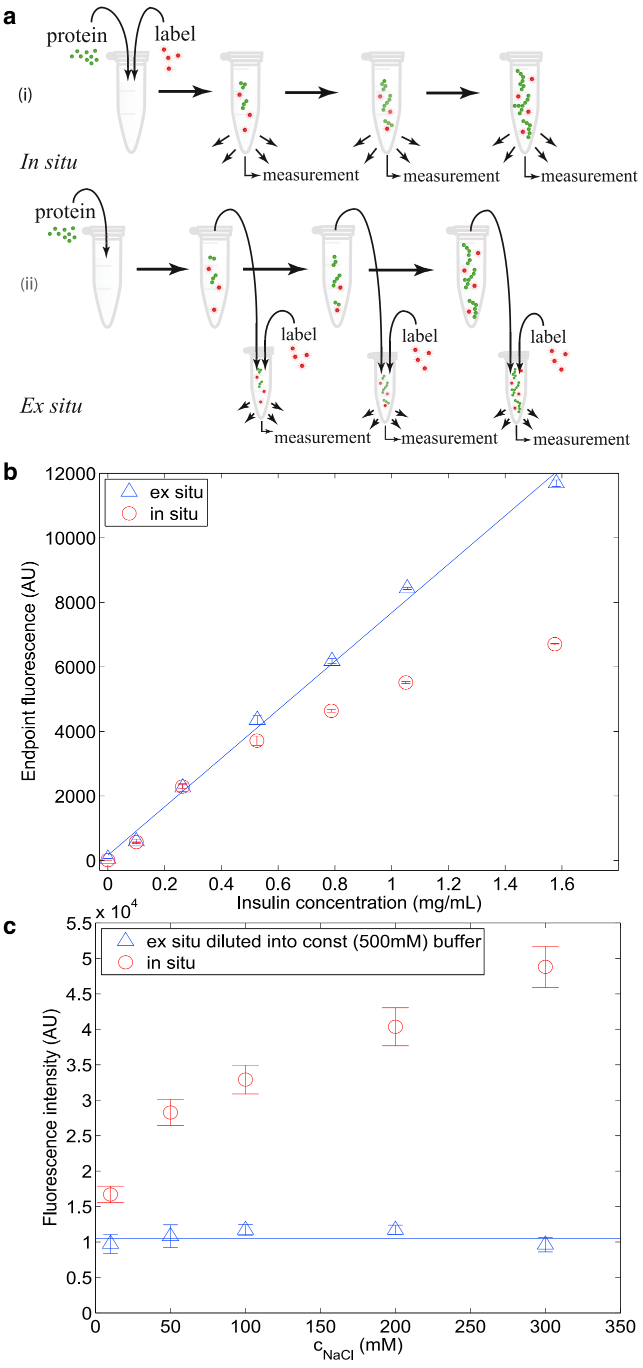

FIGURE 1 Advantages of ex situ over in situ aggregation assays. (*a*) In situ assays (*I*) are straightforward to follow but they require direct introduction of probe molecules into the aggregation assay. Interference of the aggregation process by probe molecules can be avoided in ex situ assays (*II*), where small samples of an aggregating protein solution are extracted and diluted at specified time points into a buffered solution containing an appropriate dye molecule. But conventional ex situ assays are labor-intensive and may perturb the aggregation mixture. (*b*) Fluorescence intensity of the aggregation end-point in ex situ (*blue triangles*; $R^2 = 0.997$) and in situ (*red circles*) assays at different concentrations of insulin in the same aggregation environment (50 mM NaCl, pH 2.0) recorded on a microplate reader. (*c*) When diluting ex situ assays, performed at varying salt concentrations into a set measurement solution (500 mM NaCl) containing the probe molecules, any effect of the aggregation solution can be eliminated (*blue triangles*); in the in situ assays, the conditions of the aggregation solution affect the recorded fluorescence values (*red circles*). All measurements are the average of $n = 3$ repeats. To see this figure in color, go online.

linear scaling between the fluorescence intensity and the concentration of aggregates (Fig. 1 *b*, *triangles*) and hence quantitative interpretation of the latter, the reaction and measurement steps are spatially separated and chemically decoupled, making comparison between aggregation reactions that take place in different environmental conditions (e.g., buffer) possible (Fig. 1 *c*, *triangles*).

Despite their advantages, conventional ex situ measurements (Fig. 1 *a II*) possess some drawbacks that have limited their widespread adoption: they are laborious to perform, as every time point requires manual intervention; and the reaction can be followed only at a limited number of time points. Furthermore, the requirement to physically separate aliquots from the reaction mixture implies the use of large physical volumes for the aggregating protein solution. Last but not least, withdrawing aliquots often results in agitation of the reaction mixture and can have a significant effect on aggregation if the process is to be studied under quiescent conditions.

Here we address the limitations of conventional ex situ assays by designing and fabricating microfluidic devices that automate the manual aliquot-taking process required for conventional ex situ measurements, eliminate the need to physically separate aliquots from the reaction mixture, and allow the monitoring of protein aggregation in real-time. This strategy not only decouples the aggregation process and the measurement spatially to remove any interference between the aggregation process and the probe, it also ensures that the reaction time between the dye and the aggregate species is tightly controlled and kept constant. We show that the strategy underlies a significant improvement in terms of data throughput compared to manual ex situ assays and allows hundreds of individual measurements to be performed in real-time on a single aggregating protein system.

MATERIALS AND METHODS

Fabrication of microfluidic devices

Single-layer (SL) microfluidic devices were fabricated in PDMS (poly(dimethylsiloxane); Dow Corning, Midland, MI) through single, standard soft-lithography steps using SU-8 3050 photoresist on a polished silicon wafer (25,26). The channels were sealed with a microscope glass slide after the surface had been activated with oxygen plasma.

Multilayer (ML) devices were generated by plasma-bonding two individual PDMS chips to each other (27). One of the PDMS chips was prepared in a manner analogous to the single-layer devices described above, while the other involved two subsequent UV lithography steps performed with SU-8 3025 and 3050, respectively. The side channels containing the dye as well as the channel of the mixing and labeling area (Fig. 2 *a*) were fabricated onto both microfluidic chips so that they spanned across the height of the device. The protein channel, however, was fabricated only in the second lithography step of the double-layer chip so that it would form the middle layer of the 1+2 layers.

To achieve an alignment accuracy of the order of micrometers, the two plasma-activated chips were coated in methanol and placed on top of one

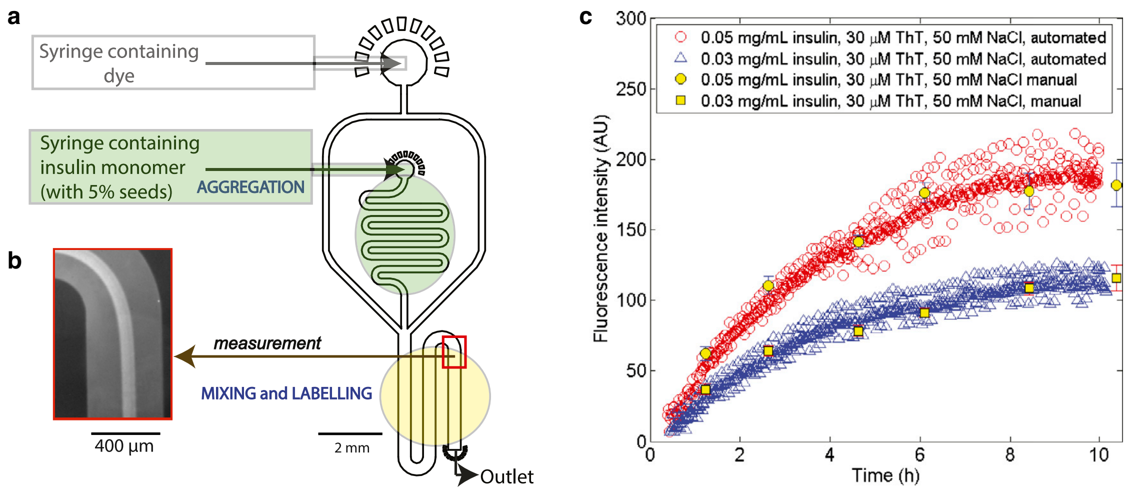

FIGURE 2 (*a*) Architecture of the SL microfluidic device used in this work. The device has two inlets, one for the solution containing the probe (dye molecule), and the other for the sample solution (contains the aggregating protein). When the streams converge, the dye can start to diffuse into the protein stream and hence bind the aggregating protein that becomes labeled (*yellow*). The fluorescence intensity is recorded further downstream where the diffusion of the dye into the protein stream has resulted in complete binding. (*b*) A fluorescence microscopy image of the measurement area. (*c*) Seeded ex situ aggregation curves obtained at 0.03 mg mL^{-1} (*blue triangles*) and 0.05 mg mL^{-1} (*red circles*) of bovine insulin using the device in (*a*) together with manually performed control assays at the same concentrations (*yellow squares* and *yellow circles*, respectively; average of $n = 3$ repeats). To see this figure in color, go online.

another, allowing for adjustment in their relative positions under a stereomicroscope (4.5× magnification). The aligned chips were then placed in an oven at 65°C for 1 h to allow evaporation of the methanol and the covalent bonding to take place. Alignment between these lithography processes was achieved through a custom-built mask aligner including an *x,y,z* and a rotating stage (cat. nos. MBT602/M and PR01/M; ThorLabs, Newton, NJ).

Fibril formation assays in microplates

All aggregation assays involved bovine insulin (cat. no. GEM-700-112-P, used without further purification; Sera Laboratories, Haywards Heath, West Sussex, UK) in a specified concentration of NaCl at pH 2.0 (adjusted with HCl). To accelerate the aggregation process, 5% of the initial insulin was added in the form of preformed seeded fibrils, and the rest of the protein was in its monomeric form. The preformed fibrils were generated by incubating 10 mg mL^{-1} insulin (pH 2, no NaCl) for 24 h at 60°C with maximum stirring. Before adding the seeds to the aggregation mixture, they were sonicated (3 times for 5 min, cat. no. HD2070, 8 W, VS 70 probe; Bandelin Sonopuls, Berlin, Germany) to fragment the fibrils and hence to increase the number of free ends available for aggregation (28). ThT (final concentration of 30 μM) was added to the aggregation assay either at the start of the aggregation process (in situ assays) or right before the fluorescence reading was taken (ex situ assays). In both cases the fluorescence intensities were recorded on a microplate reader (FLUOstar; BMG LabTech, Thermo Fisher Scientific, Waltham, MA) at 30°C using 96-well, half-area black bottom polystyrene plates (product no. 3881; Corning Life Sciences, Tewksbury, MA) with a 100 μL sample in each well. The multiwell plate was sealed with an aluminum sealing tape (product no. 6570; Corning Life Sciences) to reduce any evaporation of the fluid from the wells.

Manual ex situ assays involved forming a number of separate aliquots of the aggregation assay to be studied. ThT (final concentration of 30 μM) was added at different time points during the aggregation process, with the fluorescence intensity recorded on a microplate reader as described earlier, right after the ThT solution had been introduced.

To examine the effect of the aggregation medium on the resulting fluorescence intensity, 0.5 mg mL^{-1} bovine insulin was set to aggregate with (in situ) and without (ex situ) the addition of ThT in solutions of various ionic strengths (20–300 mM NaCl) until the aggregation had reached completion. The ex situ assays were then diluted into a set ThT solution for measurement to permit comparison between the protein concentrations. A 1:10 dilution into a ThT solution of 500 mM NaCl (pH 2) was chosen for the salt that was present in the original aggregation solution so as not to affect the recorded values.

Measurements in microfluidic devices

The solution of insulin under the aggregation conditions of interest was injected into the microfluidic device at a flow rate of 50 $\mu L\ h^{-1}$ for SL devices and 5 $\mu L\ h^{-1}$ for ML devices. Syringe pumps (neMESYS modules; Cetoni, Korbussen, Germany) were used to control the flow. ThT (30 μM) was injected from the other inlet (Fig. 2 *a*) at a flow rate of 200 $\mu L\ h^{-1}$ or 20 $\mu L\ h^{-1}$ for the SL or ML devices, respectively. The ThT solution was prepared in the same solution (50 mM NaCl at pH 2.0) as the aggregation mixture.

The fluorescent signal was recorded (Axio Observer D1 inverted microscope; Carl Zeiss, Jena, Germany and Evolve 512 EMCCD camera; PhotoMetrics, Huntington Beach, CA) at a fixed point (Fig. 2 *a*) in the mixing and labeling area (marked in *yellow*) using a 440/480 nm filter (excitation and emission wavelengths) at an exposure time of 5 s. The point chosen was sufficiently downstream for the dye to have diffused into the protein stream (seconds; Fig. 2 *b*; see the Supporting Material). Data points were collected every 1 min over a 10 h period, by the end of which the aggregation reaction had approached completion. The fluorescence intensity at each data point was calculated as the difference between the fluorescence intensity of the ThT bound to the aggregates in the center of the microfluidic channel and that of the unbound ThT at the edges of the channel (Fig. S3).

RESULTS AND DISCUSSION

Microplate reader assays

We first evaluated the performance of in situ and ex situ assays for the same protein aggregation reaction in bulk-scale experiments. To this effect, a series of different concentrations of insulin was left to aggregate for 12 h after seeding as described in Materials and Methods. The values of the fluorescence intensity for both in situ and ex situ assays

were recorded and are shown in Fig. 1 *b*. Ex situ assays exhibit linear scaling of the end-point fluorescence intensity values with insulin concentration across the range of concentrations studied (*blue triangles*); by contrast, for in situ assays, a simple linear scaling was not observed in the same aggregation solutions (*red circles*). The lack of a simple relationship between the fluorescence intensity and the concentration of aggregated protein in the in situ assays is a general trend, as we demonstrated under a variety of conditions (Fig. S2), with similar trends being observed for other systems (23,24).

We further followed in situ aggregation of a preset concentration of insulin (0.5 mg mL^{-1}) in aggregation solutions of varying the NaCl concentration; the presence of salt had a marked effect on the observed signal in the in situ assays (Fig. 1 *c*, *red circles*), making a connection between the observed fluorescence and the concentration of aggregated protein challenging to establish. By contrast, in the ex situ assay, the presence of salt did not affect the reading because the measurement was always taken at set conditions (Fig. 1 *c*, *blue triangles*).

Microfluidic ex situ measurements

The data in Fig. 1, *b* and *c*, highlight the robustness of ex situ assays under varying solution conditions. However, the use of such methods by a conventional laboratory is slow, labor-intensive, and requires large volumes of the aggregating protein mixture to allow for a number of aliquots with macroscopic volumes to be extracted. Furthermore, such ex situ measurements perturb the aggregating sample, making it virtually impossible to reliably analyze aggregation processes if quiescent conditions are required.

We thus set out to develop a microfluidic platform to automate ex situ measurements, retaining all of its advantages—most notably the spatial separation and chemical decoupling of reaction and measurement—while also offering the additional benefits of eliminating the need for manual aliquot taking, allowing tight control over the reaction time of the dye with the aggregate species, avoiding agitation of the aggregation mixture, and providing the capability to follow the process in real-time on a single chip. The objective was achieved by continuous transfer of a label-free aggregating protein sample to a measurement system containing buffered dye, with the labeling step occurring only immediately before the measurement is taken (Fig. 2 *a*). The fluid moved through the mixing and labeling zone (marked *yellow*) in a few seconds—a timescale that is very short compared to that of the studied aggregation process. Hence, effectively no further aggregation occurred in the presence of the probe molecules.

Aggregation curves obtained for 0.03 mg mL^{-1} (*blue triangles*) and 0.05 mg mL^{-1} (*red circles*) insulin assays are shown in Fig. 2 *c* (recorded at a time resolution of 60 s). The fluorescence signal, at long times when aggregation had reached completion, can be seen to be proportional to the initial monomer concentrations, as expected for ex situ measurement (113 $\pm$ 6 AU and 192 $\pm$ 11 AU for 0.03 mg mL^{-1} and 0.05 mg mL^{-1}, respectively; average of $n = 30$ final values). The observed two-level fluctuations in the data are likely to have arisen from pulsation of the flow. The aggregation curves obtained in the microfluidic devices agree with the ex situ aggregation curves obtained manually but with a throughput that is increased by approximately two orders of magnitude.

Over 12 h, this single-layer design architecture was found to yield reliable results up to protein concentrations of ~0.05 mg mL^{-1}. For higher concentrations, adhesion was observed (Fig. S4). To follow the aggregation process accurately at higher concentrations, a multilayer (ML) device (Fig. 3 *b*, *inset*) was designed and built (see Materials and Methods) that allowed the sample aliquot stream to be flanked by co-flowing labeling solution in all directions, thus preventing direct contact between the protein and the walls of the microfluidic channel where adhesion could otherwise occur.

Using such a ML device, a seeded aggregation curve, at an insulin concentration of 0.2 mg mL^{-1}, was obtained (Fig. 3 *a*), serving to illustrate that the device opens up the possibility to study aggregation at higher concentrations of protein. As for the lower concentrations, the data agree with manually performed ex situ assays where ThT molecules were introduced into separate aliquots at various time points just before the measurement was taken. In Fig. 3 *b*, the three aggregation curves obtained (0.03 mg mL^{-1} and 0.05 mg mL^{-1} for SL, and 0.2 mg mL^{-1} for a ML device) are normalized with respect to the intensity at long times (the average of $n = 30$ final values); all the curves can be seen to exhibit an absence of lag phase and to collapse to a good approximation onto a master curve, as expected for first-order kinetics observed under fully seeded conditions (29,30).

These microfluidic devices have a number of major advantages over conventional methods of following protein aggregation reaction. Most importantly, spatial separation ensures that the aggregation reaction is decoupled from the measurement process, hence eliminating the type of interference between the probe and the aggregating protein that is encountered in the in situ assays. In addition to providing such spatial separation between the two processes, the devices allow the aggregation reaction and the measurement step to be performed in chemically different environments. Thus, for example, a different buffer, such as one with higher ionic strength, could be used to prepare the ThT solution to amplify the fluorescence signal (31) but leaving the environment of the aggregation unaffected; the latter process could still take place in a buffer with a lower ionic strength. Furthermore, comparison between aggregation reactions happening in different environments is possible using a set ThT solution, as illustrated in Fig. 1 *c* (*triangles*).

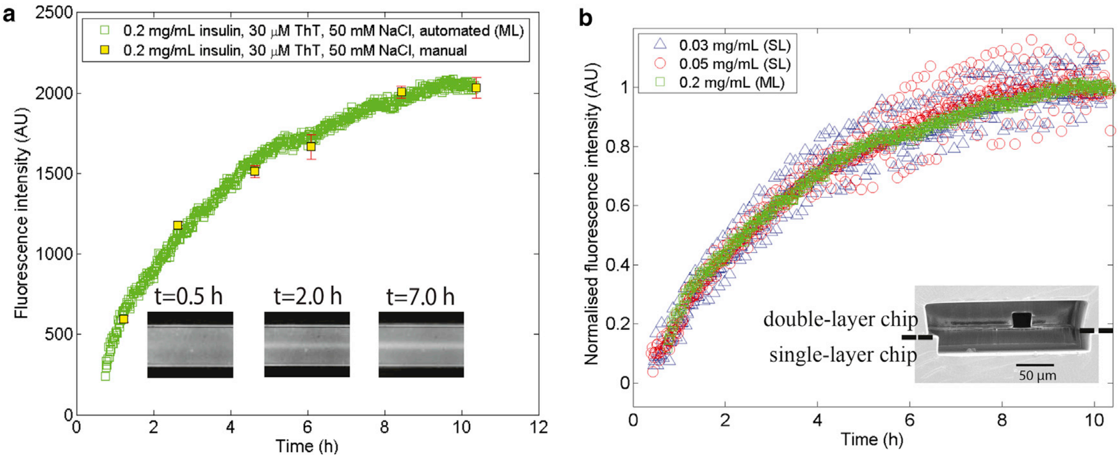

FIGURE 3 (*a*) Higher-concentration (0.2 mg mL^{-1} insulin) seeded ex situ aggregation curve, obtained with the ML device, together with manual assays (*yellow squares*, average of $n = 3$ repeats). (*b*) The three curves (two lower protein concentrations (0.03 mg mL^{-1}, *blue triangles* and 0.05 mg mL^{-1}, *red circles*) in the SL device, the highest concentration (0.2 mg mL^{-1}, *green squares*) in the ML device) normalized with respect to the average of the $n = 30$ final values. (*Inset*) Mean ± SE image of the cross section of the ML device at the point where the sample and the dye first converge. To see this figure in color, go online.

Next, the acquisition of ~600 data points over a time period of 10 h is more than an order-of-magnitude improvement over manually performed ex situ measurements of protein aggregation where the time resolution is usually of the order of tens of minutes, as defined by the normal aliquoting of a reaction cycle (32–35). The process of transferring the protein sample into the measurement system is continuous; fluid handling is no longer the limitation of the achieved time resolution. Instead, it is the detection system that determines the frequency of measurements. The outlined strategy also opens the possibility to study processes that exhibit fast kinetics and can be challenging to be followed by manual aliquot taking.

A further key feature of these assays is that the small scale of microfluidic devices combined with the lack of the requirement to physically separate macroscopic aliquots enables only miniature volumes of protein solution to be used. More specifically, the SL device consumed ~500 μL of protein sample to obtain the full ex situ aggregation curve. As the high protein flow rate is a necessary requirement to eliminate adhering of protein fibrils to the PDMS walls, this design is best suited for experiments that are less sensitive toward sample consumption. The ML device, however, is not restricted to the use of specific flow rates, because any interaction between the PDMS walls of the device and the protein is eliminated due to three-dimensional flow. In this work, ~75 μL of protein sample (includes 25 μL of dead volume connecting the syringe to the microfluidic device; this could be reduced further) was sufficient to acquire the full ex situ aggregation curve (600 data points over 10 h; 120 nL of sample per measurement point). The flow rate can be decreased further until the precision of the syringe pumps is reached. Which of the two types of devices is more appropriate for a particular experiment is a compromise between a multistep fabrication technique and the amount of protein sample consumed.

In addition to this, if following the aggregation process at high time resolution or continuously is not of relevance, the syringe pump could be operated under a flow ramp with the protein sample ejected only at the time when a measurement is to be taken or control valves could be added to the design (36,37), thus further reducing protein consumption. This could be particularly relevant for nonseeded aggregation assays where high time resolution is less beneficial during some stage of the process than it is for others. This approach is applicable to both SL and ML devices.

It is worth noting that, unlike conventional ex situ assays, this microfluidic setup does not rely on user interactions to physically extract aliquots from the aggregation mixture, thus reducing the risk of perturbing the sample and affecting the aggregation process. This is especially of import if fully quiescent aggregation conditions are required. We note that agitation could be introduced with small-scale magnetic stirring.

Last but not least, the design strategy that this device uses can be extended to other types of measurement strategies where interference with probe is not desirable.

CONCLUSIONS

Conventional ex situ measurements yield high-quality data, but are labor-intensive to perform; they can disturb the reaction mixture and allow reactions to be monitored only at limited numbers of time points, rather than continuously. To address those limitations, we have developed high throughput microfluidic devices that eliminate fluid aliquoting as the limiting factor of the time resolution of ex situ assays and instead allow continuous real-time automated measurements of such assays. Such devices open up the possibility of extension to other measurement systems, where

interference with a probe used to monitor a reaction must be avoided.

SUPPORTING MATERIAL

Supporting Materials and Methods and four figures are available at http://www.biophysj.org/biophysj/supplemental/S0006-3495(15)04707-4.

AUTHOR CONTRIBUTIONS

K.-L.S., E.V.Y., T.M., and T.P.J.K. designed research; K.-L.S., E.V.Y., T.M., and S.S. performed research; K.-L.S. and E.V.Y. analyzed the data; and K.-L.S., E.V.Y., T.M., C.M.D., and T.P.J.K. wrote the article.

ACKNOWLEDGMENTS

We thank Jeremy N. Skepper (Multi-Imaging Centre, University of Cambridge) for assistance with electron microscopy imaging.

We gratefully acknowledge financial support from the Frances and Augustus Newman Foundation, the Biotechnology and Biological Sciences Research Council, the Engineering and Physical Sciences Research Council, the European Research Council, and the Swiss National Science Foundation.

REFERENCES

1. Dobson, C. M. 2003. Protein folding and misfolding. *Nature.* 426:884–890.
2. Ross, C. A., and M. A. Poirier. 2004. Protein aggregation and neurodegenerative disease. *Nat. Med.* 10 (*Suppl*):S10–S17.
3. Cheang, M. C., M. van de Rijn, and T. O. Nielsen. 2008. Gene expression profiling of breast cancer. *Annu. Rev. Pathol.* 3:67–97.
4. Otzen, D., and P. H. Nielsen. 2008. We find them here, we find them there: functional bacterial amyloid. *Cell. Mol. Life Sci.* 65:910–927.
5. Mezzenga, R., and P. Fischer. 2013. The self-assembly, aggregation and phase transitions of food protein systems in one, two and three dimensions. *Rep. Prog. Phys.* 76:046601.
6. Li, C., J. Adamcik, and R. Mezzenga. 2012. Biodegradable nanocomposites of amyloid fibrils and graphene with shape-memory and enzyme-sensing properties. *Nat. Nanotechnol.* 7:421–427.
7. Reches, M., and E. Gazit. 2006. Controlled patterning of aligned self-assembled peptide nanotubes. *Nat. Nanotechnol.* 1:195–200.
8. Kelly, J. W. 2002. Towards an understanding of amyloidogenesis. *Nat. Struct. Biol.* 9:323–325.
9. Chiti, F., and C. M. Dobson. 2006. Protein misfolding, functional amyloid, and human disease. *Annu. Rev. Biochem.* 75:333–366.
10. Rivers, R. C., J. R. Kumita, …, J. Christodoulou. 2008. Molecular determinants of the aggregation behavior of α- and β-synuclein. *Protein Sci.* 17:887–898.
11. Roodveldt, C., J. Christodoulou, and C. M. Dobson. 2008. Immunological features of α-synuclein in Parkinson's disease. *J. Cell. Mol. Med.* 12:1820–1829.
12. Aguzzi, A., and A. M. Calella. 2009. Prions: protein aggregation and infectious diseases. *Physiol. Rev.* 89:1105–1152.
13. Lee, S.-J., P. Desplats, …, E. Masliah. 2010. Cell-to-cell transmission of non-prion protein aggregates. *Nat. Rev. Neurol.* 6:702–706.
14. Valastyan, J. S., and S. Lindquist. 2014. Mechanisms of protein-folding diseases at a glance. *Dis. Model. Mech.* 7:9–14.
15. Hawe, A., M. Sutter, and W. Jiskoot. 2008. Extrinsic fluorescent dyes as tools for protein characterization. *Pharm. Res.* 25:1487–1499.
16. Chander, H., A. Chauhan, and V. Chauhan. 2007. Binding of proteases to fibrillar amyloid-β protein and its inhibition by Congo Red. *J. Alzheimers Dis.* 12:261–269.
17. Groenning, M. 2010. Binding mode of thioflavin T and other molecular probes in the context of amyloid fibrils' current status. *J. Chem. Biol.* 3:1–18.
18. Porat, Y., A. Abramowitz, and E. Gazit. 2006. Inhibition of amyloid fibril formation by polyphenols: structural similarity and aromatic interactions as a common inhibition mechanism. *Chem. Biol. Drug Des.* 67:27–37.
19. Frydman-Marom, A., R. Shaltiel-Karyo, …, E. Gazit. 2011. The generic amyloid formation inhibition effect of a designed small aromatic β-breaking peptide. *Amyloid.* 18:119–127.
20. Buell, A. K., C. M. Dobson, …, M. E. Welland. 2010. Interactions between amyloidophilic dyes and their relevance to studies of amyloid inhibitors. *Biophys. J.* 99:3492–3497.
21. Biancalana, M., and S. Koide. 2010. Molecular mechanism of thioflavin-T binding to amyloid fibrils. *Biochim. Biophys. Acta.* 1804:1405–1412.
22. Biancalana, M., K. Makabe, …, S. Koide. 2009. Molecular mechanism of thioflavin-T binding to the surface of β-rich peptide self-assemblies. *J. Mol. Biol.* 385:1052–1063.
23. Uversky, V. N., J. Li, and A. L. Fink. 2001. Metal-triggered structural transformations, aggregation, and fibrillation of human α-synuclein. A possible molecular NK between Parkinson's disease and heavy metal exposure. *J. Biol. Chem.* 276:44284–44296.
24. Foderà, V., F. Librizzi, …, M. Leone. 2008. Secondary nucleation and accessible surface in insulin amyloid fibril formation. *J. Phys. Chem. B.* 112:3853–3858.
25. Duffy, D. C., J. C. McDonald, …, G. M. Whitesides. 1998. Rapid prototyping of microfluidic systems in poly(dimethylsiloxane). *Anal. Chem.* 70:4974–4984.
26. Qin, D., Y. Xia, and G. M. Whitesides. 2010. Soft lithography for micro- and nanoscale patterning. *Nat. Protoc.* 5:491–502.
27. Tran, T. M., S. Cater, and A. R. Abate. 2014. Coaxial flow focusing in poly(dimethylsiloxane) microfluidic devices. *Biomicrofluidics.* 8:016502.
28. Huang, Y. Y., T. P. J. Knowles, and E. M. Terentjev. 2009. Strength of nanotubes, filaments, and nanowires from sonication-induced scission. *Adv. Mater.* 21:3945–3948.
29. Cohen, S. I. A., M. Vendruscolo, …, T. P. J. Knowles. 2011. Nucleated polymerization with secondary pathways. I. Time evolution of the principal moments. *J. Chem. Phys.* 135:065105.
30. Cohen, S. I. A., M. Vendruscolo, …, T. P. J. Knowles. 2011. Nucleated polymerisation in the presence of pre-formed seed filaments. *Int. J. Mol. Sci.* 12:5844–5852.
31. LeVine, H., 3rd 1997. Stopped-flow kinetics reveal multiple phases of thioflavin T binding to Alzheimer β (1–40) amyloid fibrils. *Arch. Biochem. Biophys.* 342:306–316.
32. Hoyer, W., T. Antony, …, V. Subramaniam. 2002. Dependence of α-synuclein aggregate morphology on solution conditions. *J. Mol. Biol.* 322:383–393.
33. Hong, Y., L. Meng, …, B. Z. Tang. 2012. Monitoring and inhibition of insulin fibrillation by a small organic fluorogen with aggregation-induced emission characteristics. *J. Am. Chem. Soc.* 134:1680–1689.
34. Wang, J.-B., Y.-M. Wang, and C.-M. Zeng. 2011. Quercetin inhibits amyloid fibrillation of bovine insulin and destabilizes preformed fibrils. *Biochem. Biophys. Res. Commun.* 415:675–679.
35. Xu, L.-Q., S. Wu, …, S. Perrett. 2013. Influence of specific HSP70 domains on fibril formation of the yeast prion protein Ure2. *Philos. Trans. R. Soc. Lond. B Biol. Sci.* 368:20110410.
36. Oh, K. W., and C. H. Ahn. 2006. A review of microvalves. *J. Micromech. Microeng.* 16:R13–R39.
37. Abate, A. R., and D. A. Weitz. 2008. Single-layer membrane valves for elastomeric microfluidic devices. *Appl. Phys. Lett.* 92:243509.

Automated *ex situ* aggregation assays - Supporting Material

K.-L. Saar, E. V. Yates, T. Müller, S. Saunier, C. M. Dobson and
T. P. J. Knowles

1 Pre-formed insulin fibrils

In order to accelerate the aggregation process 5% of the initial bovine insulin was added in the form of preformed fibrils. These fibrils were generated by incubating 10 mg mL^{-1} insulin solution (pH 2, no NaCl) for 24 hours at 60 °C with maximum stirring. Before adding the thus formed seeds to the aggregation mixture, they were sonicated (3 times for 5 minutes, Bandelin Sonopuls HD2070, 8 W, VS 70 probe) in order to fragment the fibrils and hence increase the number of free ends available for aggregation. Figure S1 shows an AFM image of the preformed fibrils.

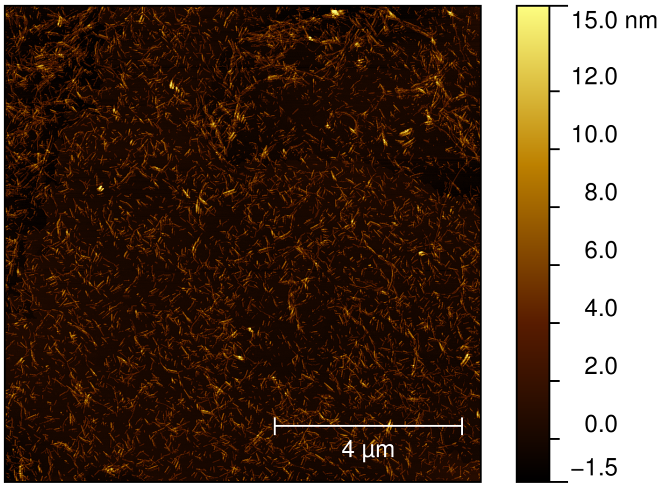

Figure S1. An AFM image of the preformed seeds that were added to the aggregation assay in order to accelerate the aggregation process.

2 Non-linear scaling in *in situ* assays

As demonstrated in Figure 1b (main text), linear scaling of fluorescence intensity with bovine insulin aggregate concentration appears to be a general trend of *ex situ* assays within the concentration range of protein studied. However, such linear scaling is not observed for *in situ* assays under the same aggregation conditions. We investigated further whether the absence of such scaling is specific to the conditions used in these experiments or a more general trend that is occurring in other of the buffer condition and other concentration of dye. Indeed, Figure S2 illustrates the absence of a simple scaling relationship between the fluorescence intensity and the endpoint concentration of bovine insulin fibrils in 50 mM and 100 mM NaCl buffers (both at pH 2.0) followed at ThT concentrations of 2 μM and 20 μM ThT.

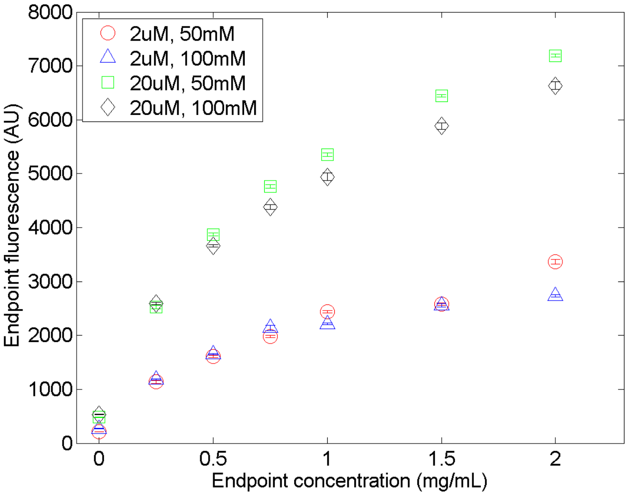

Figure S2. Absence of simple linear scaling between fluorescence intensity and insulin fibril concentration in *in situ* assays aggregating in 50 mM and 100 mM NaCl buffers (both at pH 2.0) followed at ThT concentrations of 2 μM and 20 μM. Linear scaling is observed in *ex situ* assays as demonstrated in Figure 1b of the main text of the Article.

3 Device design

In order to guide the design of the device the following calculations were performed.

By the time a section of fluid reaches the measurement point the dye should have fully diffused into the protein stream. For example, in an SL device, ThT needs to diffuse by

$$x = \frac{1}{2} \times w_{protein} = \frac{1}{2} \times w_{total} \times \frac{Q_{protein}}{Q_{total}} = 0.5 \times 400\mu m \times \frac{50\ \mu L\ h^{-1}}{(200+50)\ \mu L\ h^{-1}} = 40\mu m \quad (1)$$

where the factor $\frac{1}{2}$ arises from symmetry and $w_{protein}$ and w_{total} represent the width of the protein stream in the measurement area and that of the measurement channel, respectively. Q_i's correspond to the respective flow rates.

Hence, it takes

$$t_{ThT} \sim \left(\frac{x^2}{2 \times D_{ThT}}\right) \sim \left(\frac{(40 \times 10^{-6})^2\ m^2}{2 \times 8 \times 10^{-10}\ m^2\ s^{-1}}\right) \sim 1s \quad (2)$$

for the ThT to diffuse into the protein stream.

Finally, in a rectangular duct the centreline velocity of the fluid can be approximated to be:

$$u_{CL} = \frac{Q_{total}}{A} = \frac{\frac{250\ mm^3}{h} \times \frac{1\ h}{3600\ s}}{400 \times 10^{-3}\ mm \times 100 \times 10^{-3}\ mm} = 1.7\ mm\ s^{-1} \quad (3)$$

The measurement was taken around 14 mm downstream of the point where the fluids first mix to ensure complete mixing of the ThT molecules with the fibrils.

4 Fluorescence intensity measurements and calculations

As explained in the previous section the fluorescence intensity at each time point was recorded at a point sufficiently downstream for the ThT molecules to have fully diffused into the protein stream. The recorded values were calculated as the difference between the fluorescence intensity of the ThT bound to fibrils (centre of the channel; the average value across the blue rectangle with area of 600×30 pixels) and that of the non-bound form (edges of the channel, the average value across the two brown rectangles each with area of 600×200 pixels) as illustrated in Figure S3. Averaging across the rectangular shapes allows eliminating any possible effects that could have arisen from non-uniform illumination of the field of view.

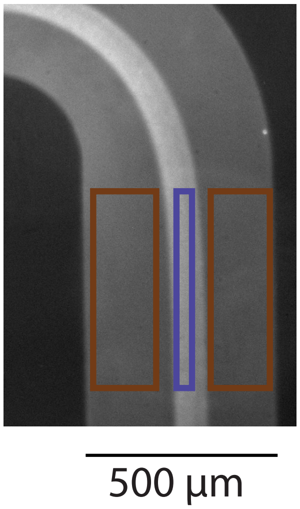

Figure S3. The fluorescence intensity at each time point was calculated as the difference between the fibril-bound form of the ThT (centre of the channel) and its non-bound form (edges of the channel). Both values were averaged across rectangular shapes in order to eliminate any variations from potential non-uniform illumination of the imaging area.

5 Adhesion in SL devices at higher concentrations of protein

The SL device could be effectively used for following protein aggregation at lower protein concentrations. However, at higher concentrations adhesion of the protein to the PDMS surface was observed. For instance, Figure S4 shows the adhesion in a SL device at protein fibril concentration of 0.2 mg mL^{-1} after 2 hours. Hence, ML devices were design and fabricated to

eliminate any interaction between protein stream and the walls of the microfluidic channel by flaking the protein stream by co-flowing buffer from all directions (see main text for details).

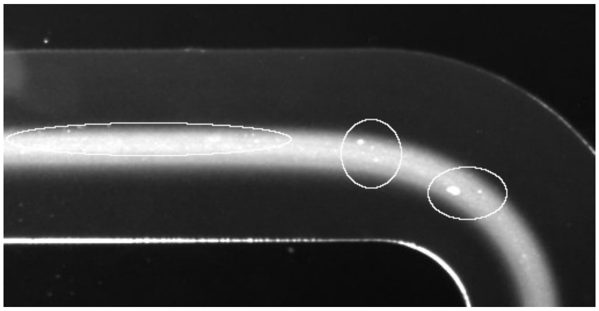

Figure S4. Fibril sticking to the PDMS surface of the channels at protein concentration of 0.2 mg mL^{-1} after 2 hours.
